# Exploring the Experiences of First Nations Caregivers of Autistic Children in Canada: A Qualitative Community-Based Participatory Research Study

**DOI:** 10.1177/13623613261464661

**Published:** 2026-07-28

**Authors:** Grant Bruno, Mariam Ahmad, Carmella Cutknife, Heather Littlechild, Trina Ertman, Jacqueline Smith, Lonnie Zwaigenbaum, David Nicholas

**Affiliations:** 1 Samson Cree Nation (Treaty 6 Territory), Maskwacîs, Alberta, Canada; 2 University of Alberta, Edmonton, Canada; 3 Women and Children’s Health Research Institute, Edmonton, Alberta, Canada; 4 Maskwacîs Parents Place, Alberta, Canada; 5 Maskwacîs Education Schools Commission, Alberta, Canada; 6 Six Nations of the Grand River, Ohsweken, Ontario, Canada; 7 University of Calgary, Alberta, Canada

**Keywords:** Indigenous, First Nation, Canada, autism, caregiver, community-based participatory research, neurodiversity

## Abstract

Autism exists across all populations, including among Indigenous Peoples, yet the experiences of First Nations caregivers of Autistic children remain significantly under-researched. This study explores the lived realities of caregivers in two First Nations communities: Maskwacîs (Alberta) and Six Nations of the Grand River (Ontario). Guided by a Community-Based Participatory Research (CBPR) approach and grounded in the nêhiyaw concept of wâhkôtowin (relationality), this research was co-led by the Autism Community Research Circle, which included Elders, Autistic individuals, caregivers, and professionals. Fourteen caregivers participated in semi-structured interviews, and thematic analysis revealed eight key themes: lack of understanding and recognition; caregiver burnout; challenges with diagnosis and clinical navigation; school experiences; advocacy; stigma and ableism; culture and ceremony; and acceptance and transformation. Caregivers identified barriers including jurisdictional divides, racism, and lack of culturally appropriate services, but also emphasized the importance of kinship, cultural identity, and community acceptance. Autism was often reframed through Indigenous worldviews as a gift from the Creator, highlighting a strength-based perspective rooted in relationality. This study addresses a critical gap in the literature and calls for culturally grounded, community-led autism supports that align with Indigenous knowledge systems. Findings offer essential insights to inform more responsive policy, programming, and future research.

**Lay Abstract:**

Autism exists in every community, including among First Nations Peoples in Canada, yet very little research has explored the experiences of Indigenous families raising Autistic children. This study looked at what life is like for First Nations caregivers of Autistic children in two communities, Maskwacîs in Alberta and Six Nations of the Grand River in Ontario. The project was led in partnership with community members, including Autistic people, Elders, caregivers, and professionals, through the Autism Community Research Circle. Together, we used a community-based approach that followed local teachings and values, especially the Cree concept of wâhkôtowin, which means kinship and relationship. Fourteen caregivers took part in interviews where they shared their stories and perspectives. We learned that caregivers often face major challenges in getting autism diagnoses and finding appropriate supports and services. Many spoke about burnout, stigma, and racism, as well as frustration with school systems and health care providers. Despite these barriers, caregivers described the strength they draw from family, culture, and community. Cultural teachings, ceremonies, and language helped caregivers and their children feel proud, connected, and supported. Some families described autism as a gift from the Creator, showing how Indigenous ways of understanding autism can promote acceptance and belonging. This research shows the need for autism programs, services, and policies that are guided by Indigenous knowledge, community leadership, and cultural safety. It also highlights the importance of seeing autism through a strengths-based lens that values family relationships, community, and identity. By listening to First Nations caregivers, we can build more inclusive and culturally grounded supports for Autistic children and their families.

## Background

It is now widely recognized that autism exists across all populations, including among Indigenous Peoples (Bailey & Arciuli, 2020; Gerlach et al., 2022; Inman, 2019; Simpson, 2021; Tupou et al., 2021). However, Indigenous-specific autism research remains limited and is often shaped by Western biomedical and service-access frameworks rather than Indigenous epistemologies. Existing studies with Indigenous Peoples in Australia, Māori communities, and Indigenous Peoples in Canada identify recurring concerns related to under-recognition, misdiagnosis, service barriers, cultural identity, and the need for Indigenous-led approaches that center family, language, community knowledge, and self-determination (Bailey & Arciuli, 2020; Inman, 2019; Simpson, 2021; Tupou et al., 2021). These gaps are especially pronounced in relation to the diagnostic, cultural, and systemic realities faced by Indigenous families (Antony et al., 2022; Lindblom, 2014).

Canada’s Constitution Act of 1982 recognizes three distinct Indigenous groups: First Nations, Métis, and Inuit. Together, they represent approximately 1.8 million people, or about 5% of the national population (Statistics Canada, 2022). Since Confederation, Canada has enacted and maintained systemic racism against Indigenous Peoples, embedded within both historical and contemporary policies and legislation. One prominent example is the Indian Act of 1876, which continues to regulate many aspects of First Nations life (Bartlett, 1977). In the context of autism and disability, this legislation contributes to an intersection of structural ableism and racism by reinforcing jurisdictional divides: while reserves fall under federal responsibility, disability services (e.g., clinical autism supports) are administered provincially (Johnson, 2018). This division has resulted in significant service gaps, as federal and provincial governments frequently dispute responsibility, often to the detriment of Indigenous families seeking support for Autistic children (Antony et al., 2022). These jurisdictional divisions shape whether families can obtain assessments, therapies, school-based supports, transportation, respite, and culturally safe services in a timely and coordinated way.

Despite growing recognition of the importance of culturally responsive care in autism research and practice, there remains a significant lack of literature examining the experiences of Indigenous caregivers of Autistic children. The limited available research suggests that Indigenous caregivers face complex barriers, including jurisdictional gaps, systemic racism, and the absence of culturally grounded autism services, all of which impact their access to diagnosis, support, and care pathways (Gerlach et al., 2022). Furthermore, Indigenous understandings of neurodiversity, kinship, and relational responsibility are rarely acknowledged, leading to interventions that may not align with community values or worldviews (Bruno et al., 2025). In many service systems, autism continues to be framed primarily through deficit, impairment, and intervention models. These models are used for accessing supports, but they can conflict with Indigenous teachings that emphasize relationality, spiritual identity, belonging, and the responsibilities of family and community to uphold the child. Addressing this critical gap requires research that is Indigenous-led, relationally accountable, and rooted in methodologies that honor Indigenous knowledge systems and caregiving practices. Autism supports in Canada are fragmented across health, education, disability, child and family services, and federal and provincial funding systems. For First Nations families, these barriers are often intensified by on-reserve service gaps, travel for diagnosis, racism in health care and education systems, and providers’ limited knowledge of First Nations histories, protocols, and cultural safety. Strengthening supports requires community-led policy, culturally grounded practice, Indigenous governance, and service pathways that recognize autism-related needs and Indigenous rights to self-determination.

## Terminology

For the purpose of this study, we intentionally use language that aligns with current practices and preferences in both Indigenous and autism focused spaces. Terms such as First Nations are used to specifically refer to the Indigenous communities who participated in this research, reflecting their distinct political and cultural identities within the Canadian context. We also use Autistic as an identity-first term, in recognition of growing advocacy within the autism community that emphasizes autism as an integral part of a person’s identity rather than something separate or pathological.

## Research Question

With full input and guidance from both Maskwacîs in Alberta and the Six Nations of the Grand River in Ontario, the research advisory collaboratively co-developed the research question: “What is the lived experience of First Nations caregivers of Autistic children and youth?”

## Methods

### Ethics

We obtained ethics approval from the University of Alberta Research Ethics Board (Pro00114136) and adhered to the Tri-Council Policy Statement 2, Chapter 9: Research Involving the First Nations, Inuit and Métis Peoples of Canada (Canadian Institutes of Health Research, 2018). Ethics and research approval for this study were obtained from the Six Nations of the Grand River (SNGR) Research Ethics Committee. The SNGR Research Ethics Committee has the authority to review and authorize all research conducted within the Six Nations territory to ensure alignment with the SNGR Research Ethics Policy and the principles of OCAP® (Ownership, Control, Access, and Possession). Approval was granted following a review of the project’s adherence to Onkwehón: we values, cultural protocols, and its demonstrated commitment to respectful community engagement and reciprocal benefit. Specific to this research, the research (GB, LZ, DN, HL, CC, TE) team attended several ceremonies in Maskwacîs and each time offered protocol to ask for guidance and support throughout the research journey. These ceremonies offered a sacred foundation to operate from and required a sacred ethical responsibility connected to the community, Ancestors, and Creator.

#### Community-Based Participatory Research

Community-Based Participatory Research (CBPR) is increasingly becoming a recognized and respected approach for conducting ethical, inclusive, and culturally grounded research with Indigenous communities (Israel et al., 2013; LaVeaux & Christopher, 2009; Tobias et al., 2013). This study was done in full partnership with Maskwacîs and the Six Nations of the Grand River. Located in Alberta, within Treaty 6 Territory, Maskwacîs is a nêhiyaw (Plains Cree) community. It is considered a large First Nation (approximately 20,000 members), and comprises four First Nations, Samson Cree Nation, Ermineskin Cree Nation, Louis Bull Tribe, and Montana First Nation. The Six Nations of the Grand River is also considered a sizable First Nation (approximately 27,000 members) and is a Haudenosaunee (Iroquois) community in Southern Ontario that is located approximately an hour and half drive southwest of Toronto. The Six Nations include the Mohawks, Senecas, Onondagas, Cayugas, Oneidas, and Tuscarora reflects the coming together of the Confederacy of the Six Nations(Weaver, 1978). Specifically, we collaborated with the Maskwacîs Education School Commission (MESC) and the Six Nations Child and Youth Health Team. Early in the research process, we obtained full MESC board and Six Nations Chief and Council approval and entered into formal research agreements, respectively. Following these approvals, we established the Autism Community Research Circle (ACRC). The ACRC met monthly and was comprised of individuals from both communities, including Autistic people, caregivers, Elders, clinical practitioners, educators, support staff, and researchers. The ACRC led the entire research project from the development of the research question and scope, providing feedback on the interview guides, participant recruitment, data analysis, and coordinating knowledge translation and research dissemination activities.

In practice, CBPR was operationalized through shared decision-making across the research process. During study design, the ACRC helped identify priorities, refine the research question, determine participant groups, and ensure the interview guide reflected community concerns. During recruitment, ACRC members advised on respectful approaches to sharing study information through community networks and trusted service relationships. During data analysis, the research team brought emerging interpretations back to the ACRC for discussion, correction, and refinement, ensuring themes were interpreted within relational, cultural, historical, and service-system contexts. During knowledge translation, the ACRC guided how findings would be returned to participating communities and shared with service providers, policymakers, and broader autism and Indigenous health audiences. This shared governance positioned community members as co-leaders, supported cultural safety, honored local protocols, and kept the research accountable to the caregivers and children whose experiences shaped the study.

### Positionality Statement

As the lead author, a community member and insider researcher, I GB acknowledge that my positionality brings both strengths and limitations to this work. Insider research can be described as when an investigator researches themselves, their family or their community (Greene, 2014; Wilkinson & Kitzinger, 2013). My lived experience, cultural grounding, and relational ties to the community offer valuable insights that enhance the relevance, trustworthiness, and relational integrity of the research. At the same time, the research team remained critically aware of the potential for bias, including the risk of overlooking taken-for-granted assumptions or unintentionally privileging certain voices over others. To address this, we engaged in ongoing reflexivity, sought guidance from Elders and knowledge keepers, and upheld relational accountability by ensuring that research processes and outcomes are guided by community priorities, ethical frameworks such as OCAP®, and Indigenous ways of knowing, being, and doing. This approach reflects a commitment to conducting research that aligns with local Indigenous worldviews, and one that is reciprocal, respectful, and grounded in collective responsibility.

## Integrating Western and Indigenous Research Methodologies

### Research Design

This study employed a qualitative description approach, guided by the nêhiyaw (Plains Cree) principle of wâhkôtowin, which emphasizes kinship, relationality, and the responsibilities that arise from our interconnectedness (Napier & Whiskeyjack, 2021; Wildcat, 2018). Qualitative description is well-suited for research seeking to explore under-examined experiences in everyday language, offering a participant-driven account that stays close to the meanings and realities shared by community members (Bradshaw et al., 2017). In alignment with wâhkôtowin, this approach foregrounds relational accountability and honors the voices and experiences of First Nations caregivers. Rather than imposing external interpretations, qualitative description, when integrated with wâhkôtowin, creates space for ethical engagement, cultural relevance, and knowledge that is grounded in relationships. This made it particularly appropriate for exploring the lived experiences of First Nations caregivers of Autistic children and youth, supporting a process that was methodologically sound and spiritually and relationally respectful.

### Data Collection

With guidance from the ACRC, we used purposeful sampling to recruit caregivers of Autistic children and youth aged from 0 to 17 years, which allowed for a rich discussion on the topic at hand (Palinkas et al., 2015). Participants were eligible if they self-identified as caregivers of an Autistic child or youth connected to one of the two participating First Nations communities. Recruitment was supported by the ACRC and community partners through trusted community networks, service contacts, and word of mouth. This relational recruitment strategy was important because of the historical and ongoing harms associated with extractive research in Indigenous communities and the sensitivity of discussing autism, disability, caregiving, and child-service systems.

Potential participants received information about the study purpose, voluntary participation, confidentiality, and their right to withdraw. Informed consent was obtained before interviews began. Participants were also reminded that they could decline to answer any question and could pause or end the interview at any time. Semi-structured interviews were conducted to support reciprocity, relational conversation, and flexibility during the interview process (Kallio et al., 2016). We conducted interviews until we reached saturation or when further data collection provided no new insights. Interviews were conducted in person and online, lasted approximately 60 min, and were conducted by research team members familiar with community protocols, confidentiality, trauma-informed interviewing, and autism-affirming language. All interviews were audio recorded, transcribed verbatim by [removed for anonymization peer review], and coded/managed with the support of ATLAS.ti data management and analysis software.

### Data Analysis and Rigor

Thematic analysis was used to interpret the data collected. This flexible and rigorous qualitative method allowed for the identification, organization and interpretation of patterns or themes (Clarke & Braun, 2014). The research team followed a recursive thematic analysis process that included transcript familiarization, initial coding, clustering related codes into candidate themes, reviewing themes against the data, and refining final themes in relation to the research question and wâhkôtowin. Codes captured key areas of caregivers’ accounts, including service navigation, diagnosis, schooling, advocacy, burnout, stigma, racism, cultural connection, ceremony, and acceptance. Analysis prioritized caregivers’ words while situating their experiences within broader structural and cultural contexts. Consistent with CBPR and wâhkôtowin, emerging themes were discussed with the ACRC to ensure interpretations resonated with community realities, reflected relevant cultural and service-system contexts, and used respectful language. This process refined the final eight themes and supported credibility, confirmability, and cultural relevance. Thematic analysis, used alongside wâhkôtowin and qualitative description, supported a culturally grounded interpretation of both shared and divergent caregiver experiences. To enhance rigor, the ACRC provided feedback on emerging learnings during the analysis phase. The findings were also presented to the MESC board and at the first annual Maskwacîs Autism Gathering and in the Six Nations of the Grand River at a community autism focused gathering to allow for their feedback. These practices functioned as community-oriented validation processes.

### Participants

We interviewed a total of 14 caregivers (demographics summarized in Table 1). This section presents a thematic analysis of caregiver interviews, identifying key themes across experiences shared by participants from Maskwacîs and Six Nations. Due to the small community size and risk of being able to identify participants, we did not collect demographic data such as age, education level, or other potentially identifying characteristics. This decision protected confidentiality in close-knit communities where combinations of demographic details could make participants recognizable.

**Table 1.** Caregiver Characteristics for *Maskwacîs* and *Six Nations* Participants (*N* = 14).

Characteristic	n	%
**Community**		
Maskwacîs	8	57
Six Nations of the Grand River	6	43
**Age of the child**		
1–6 years	4	29
7–12 years	8	57
13–17 years	2	14
**Gender of caregiver**		
Woman	13	93
Man	1	7
**Sex of child**		
Girl	0	0
Boy	14	100
**Relationship status**		
Married or common-law	4	29
Single	10	71
**Residence**		
On reserve	9	64
Off reserve	5	36

Percentages are rounded. “*n*” refers to the number of caregivers.

Terms reflect how items were recorded, specifically caregiver gender and child sex.

## Results

The findings of this study reflect the complex and multidimensional realities experienced by Indigenous caregivers of Autistic children, highlighting both shared and distinct elements across the participating communities. Eight core themes emerged from the interviews: (1) lack of understanding and recognition; (2) caregiver burnout and daily living challenges; (3) diagnostic and clinical service navigation; (4) education and school experiences; (5) advocacy and caregiver expertise; (6) stigma, discrimination, and ableism; (7) culture, language, and ceremony; (8) acceptance and transformation. Together, these themes provide insights into the structural barriers, relational dynamics, and cultural strengths that shape the Indigenous caregiving experience. Importantly, comparative insights between the Maskwacîs and Six Nations communities revealed both similarities and differences in service access, cultural frameworks, and caregiving contexts.

### Lack of Understanding and Recognition

Caregivers commonly described widespread misunderstanding of autism within healthcare, education, and broader community settings. Stereotypes about autism, such as associating it solely with a lack of affection, were identified as barriers to early recognition and support. Several caregivers noted that validation from professionals was rare but deeply impactful. Caregivers often talked about a lack of understanding of autism. They expressed the need for more awareness of autism in educational and healthcare systems and in the community overall. One caregiver who had a background as a healthcare professional discussed,

[I] had very little training in autism and when my son was showing signs, like, I would go to my colleagues and say, you know, do you think this is autism? My son is doing this, and there was a very stereotypical view of it still, where it was like, “Oh, does he hug you? Oh, no, he doesn’t have autism. If he hugs you, he doesn’t have autism.” (P04)

Caregivers also felt that such stereotypes often create barriers for Autistic children to participate fully in society and reflect presumptions among those who do not understand the reality of autism and, in turn, make misjudgments based on stereotypical behaviors such as being disruptive or inability to listen or take direction.

### Caregiver Burnout and Daily Living Challenges

Participants detailed the physical and emotional toll of caregiving, often likening it to the unrelenting demands of caring for a newborn. Mental health challenges, including exhaustion and depression, were frequently reported. The presence or absence of kinship and familial support significantly shaped caregiver well-being. Caregiver burnout was shaped by individual stress, kinship networks, community relationships, schools, clinical services, and accessible respite. Several caregivers described responsibilities that extended beyond everyday parenting, including coordinating appointments, advocating in schools, translating professional language for family members, responding to stigma, and protecting their children from racism and ableism. These responsibilities intersected with many First Nations values of relational obligation and collective care, creating sources of strength and emotional labor when formal systems failed to support families adequately.

Caregivers shared their experiences on how challenging providing care to an Autistic child can be and felt strongly that unless one has experience with providing care to an Autistic child, they would have difficulty fully understanding the experience,

It’s hard for people to kind of understand, and I’ve experienced it myself going from a parent, like parenting a neurotypical child, and parenting a child with autism. I see the difference, and I see the challenges, and I fully believe that you will not understand what it’s like to parent a child with autism until you’re parenting a child with autism. (P08)

Daily routines were described as complex and unpredictable. Common difficulties included “food selectivity”, “aversion to public spaces”, and “resistance to school attendance”. These challenges frequently led to isolation and disrupted daily functioning. One caregiver spoke about the challenges of getting their child to school every day and keeping them motivated to keep attending, stating, “where I’m struggling is he doesn’t want to go to school. I don’t know, I don’t know what other stops to pull out to try and get him back into school.” One caregiver talked about their child’s restrictive eating, and how

[F]ood is something else too. He’s super, super, super fussy that he will not eat. It’s very hard for us to find something that he would feel just ate a lot like, soups or something he can eat, but others some of the other stuff he just can’t eat or won’t eat.

### Diagnostic and Clinical Service Access

While a formal diagnosis was seen as critical for accessing services and understanding the child, caregivers noted substantial diagnostic assessment and service delays and obstacles. Caregivers conveyed what they viewed as the importance of an autism assessment and getting a formal diagnosis so that their child can access services and to better understand their child. One caregiver spoke about how they “want to know every diagnosis . . . so they can help them,” and while the diagnosis helped caregivers make sense of their child’s behaviors, it also created more questions, such as “what does the future hold for her or him? What does it hold for me as a parent? How involved am I going to have to be in his life? Will they ever live independently?” Caregivers also felt that the professionals who were responsible for helping them obtain a diagnosis could be a barrier. A caregiver shared,

Our family doctor at the time told us to wait. [They] said, “you know, basically, that autism doesn’t really present until preschool” and I’m like, “no, it’s presenting now. Like I need to get him a sense that we can do what we need to do for him.” And thankfully, she agreed with me. She gave me the referral over to [the assessment clinic] and he was assessed and diagnosed I want to say in like, maybe 20 minutes with [the pediatrician], like it didn’t take long at all. (P09)

Caregivers often expressed appreciation for their clinical service providers, stating, “They gave us a lot of really good strategies and techniques.” They described sometimes being referred to other providers, for example, being “recommend[ed] because of [the child’s] noise sensitivities that [they] take them to an OT.” Another positive example shared by a caregiver was with a speech therapist and how they “did a really good job of teaching him to communicate without words . . . through alternative ways of communicating.” Yet not all experiences were positive though as one caregiver shared, “I don’t access ABA anymore because it was too oppressive,” and later shared, “I feel like the way they talk in ABA therapy is rude. They talk condescendingly, and they talk down to the kid in [a] very commanding way.” Diagnostic and clinical services were shaped by cultural safety, respect for child autonomy, and recognition of caregiver expertise. Services were helpful when providers listened and adapted strategies, but harmful when they reproduced ableist or colonial dynamics that treated children as problems to be controlled rather than whole people embedded in family, culture, and community.

The caregivers identified racism from service providers when accessing clinical services. For instance, a caregiver reflected on an experience in which a service provider was “freaked out coming here [to the reserve], just from probably the stereotypes”. Caregivers shared how important it is to have Indigenous service providers in the community. One caregiver reflected on how Indigenous teachings and cultural knowledge can be beneficial to the children,

Both [the service providers and] parents are Indigenous, and she was so respectful to him. I told her like our teachings about kids and how I expect him to be treated. Like he’s in charge of his own life. And he has a right to like his comfortability and his stress level and so I would tell her that I want you to teach him within his limits. I want you to be respectful to him, and for a year he had her and he just did so good. He learned so much in that year. (P14)

### Education and School Experiences

All caregivers discussed school-related experiences. These ranged from struggles with transitions and bullying to successful partnerships with supportive teachers. Cultural immersion programs in Six Nations and traditional values in Maskwacîs were both viewed positively by each community, although inclusion in these settings remained inconsistent. Schooling was discussed by all the caregivers at some point in their interviews. These discussions included topics such as interactions with teachers and administrative staff, learning challenges and strengths, and concerns with bullying. Caregivers spoke often about the challenges they faced when their children started school. For instance, a caregiver shared, “The first week was kind of difficult for him . . . he was only there for 3 days, he had a hard time transitioning to the school environment.” Caregivers also talked about some of the solutions that had helped with transitioning, such as “talking about when things are going to change” or “if the teacher comes out and gets him, it is a lot better.” Caregivers also felt that open lines of communication between themselves and educators are essential to making sure their children can more fully embrace their educational journeys,

I think the way I can see the school helping families is through communication. I feel like there has to be a consistent form of communication that can be updated frequently. So, meaning that if there’s something going on at home, that’s going to affect the child in the school, that there’s a way for the parent to communicate that in a time, manner, to be able to help support the school and the same thing with the school to have like regular updates for the family, so they know what’s going on. (P01)

A key concern brought up by the caregivers was bullying in schools. Caregivers were often “worried” or “concerned” that their child would be bullied. A caregiver reflected on their own childhood experience and remembered, “watching [another student] get so severely bullied in school for his difference that other kids just wouldn’t have empathy.” Caregivers expressed that the school environment can be the difference between their children living (or not living) a good quality of life, and how important it is for the teachers and classmates to “understand them enough” and “have the support” to be able to be included fully. Caregivers offered some practical tips to school staff to support Autistic students and encouraged “letting them learn at their own pace” and having more “on the land learning” opportunities.

### Advocacy and Expertise

Caregivers positioned themselves as primary advocates, often out of necessity. While some found strength in this role, others reported fatigue and frustration. A caregiver described having to become “the biggest advocate because nobody else will be. Nobody knows my child like I do.” Another caregiver reflected on a time when they were advocating for their child with a teacher who had a loud voice when teaching but refused to change their approach and they explained to the teacher, “you might be an expert in education, but I am an expert on him and he can’t handle when you raise your voice, it makes him nervous, so we ended up talking to the principal about [the situation].” The belief that the caregiver knows best was brought up again by another caregiver,

[Don’t] give up. Fight the good fight. Advocate. Like I, the one thing I will say is, you know your child best as a parent. So, if someone’s telling you something, and it’s not sitting right, then it’s okay to advocate and get a second opinion. (P07)

Caregivers noted that those working with Autistic children sometimes do not have enough knowledge about autism, and this created a barrier for them. A caregiver shared that they had “to be the biggest advocate and had to push for school testing” because the school wanted to start him at a grade level he was not ready for. While advocacy was acknowledged as important, some caregivers felt that they were too “overwhelmed” or “exhausted” to fight anymore, and without proper support, they could not advocate effectively on behalf of their children.

### Stigma, Discrimination, and Ableism

Experiences of racism, ableism, and social stigma were widely discussed among caregivers. Discrimination occurred both within and outside of community settings, with caregivers identifying a need for public education to reduce prejudice and misinformation. A caregiver shared how they are followed around [the grocery store] due to the fact that they are “visible Indigenous” and were told to “watch their children” by the security. Another caregiver shared that at a [large department store], they catch people “staring” at their children and how uncomfortable it makes them. Another caregiver shared a story when they went to eat supper at [a restaurant], and there was a table “of Caucasian people and they’re glaring at us like we don’t belong here.”

Caregivers also spoke about the stigma they anticipated once they got a diagnosis and how, “when we first got the diagnosis, we were scared and I think we were probably really kind of letting our past experience or what society says autism is, kind of fuel what our fear was, like how our how we were afraid of the diagnosis,” and also, “if autism is something to be afraid of, then it is going to negatively impact the family.” A caregiver also shared how they were misinformed and as such, used to believe, “if I just get him help, he won’t be Autistic forever.” These types of ableist beliefs were identified as present in the community as well. A caregiver shared,

Unfortunately, like, as accepting as our people are, I do see some ableism in our communities that we can address as well. You know, around not only autism, but disabilities in general, where it’s just people who aren’t thinking like that quite yet.

When it comes to attitudes and beliefs toward autism in the community, the caregivers indicated that if we can teach children at a young age what autism is and why Autistic individuals sometimes need extra support, then they will “be fine with it.”

Finally, caregivers discussed the differences in services on and off reserve and how government policies are often barriers to getting support in the community. A caregiver shared that since “we moved to the city, we are able to get all kinds of services she wouldn’t have been able to get if they were living on reserve.” Another caregiver shared the same sentiment, “We have never had any programs down here on our reserve for him.” Yet caregivers felt that living in the city for support requires them to leave kinship support, and “our family support in [the] city is limited. So, we don’t have a lot of family support, and I feel like for the last 5 years, we have been kind of doing this challenge on our own.”

### Culture, Language, and Ceremony

Cultural connection was highly valued, yet often difficult to maintain due to sensory sensitivities and a lack of inclusive programming. Culture was central to identity, belonging, regulation, learning, and wellness. Caregivers described cultural strengths such as drumming, dancing, school-based cultural activities, and helping roles such as oskâpewis. These accounts show the need for accessible cultural spaces where sensory needs, movement, communication differences, and flexible participation are understood as part of belonging rather than barriers to inclusion. Caregivers noted children’s interest in ceremony, drumming, and language, emphasizing the need for culturally grounded autism supports. Caregivers wanted to keep their children connected to the culture and in the community despite them being on the autism spectrum, but they described many challenges in doing so,

I go to the longhouse. I only took him once and it is very overwhelming for him. To sit there and to be around all these people, you know, and the singing and dancing was too much for him. Like I had to leave halfway through the ceremony with him because there’s too much for him. He’s very sensitive to loud noises. He’ll cover his ears and get mad. So, it was so hard, but I do plan on taking him to the next ceremony. (P11)

A caregiver also shared that they would like to attend other cultural events, such as powwows, but their children simply did not want to go or were “overwhelmed with the sounds and crowds.” Caregivers conveyed the importance of taking into consideration their children’s sensory challenges when planning on attending ceremonies or cultural events. Unfortunately, caregivers often would not attend due to those challenges being prohibitive.

Caregivers also noticed that there were certain cultural activities their children like to do. A caregiver shared, “He loves hunting, and he loves being on an oskâpewis (ceremony helper) for different things.” Another caregiver talked about how their child “loves the drum, and he loves dancing.” Smudging was also brought up, and several caregivers spoke about how their children “like the smell of sweetgrass.”

Cultural activities were described to meaningfully engage Autistic children in the school setting: “he loves drumming at school . . . I make sure I get to school before 8:30 am because that’s when he has a good day, which is when he gets to be with the drum.”

### Acceptance and Transformation

Caregivers shared transformative moments following a diagnosis, moving from grief to acceptance. Embracing autism as part of their child’s identity was described as essential. Cultural perspectives, particularly in Maskwacîs, framed autism as a gift, fostering more positive and resilient caregiving mindsets. Acceptance of autism was discussed frequently, and caregivers recognized the importance of acceptance when it comes to feeling supported in the community and by their families. A caregiver pointed out a difference of attitudes they feel happens in and out of the community: “I feel like in the community, we have a lot of acceptance, but outside of the community, like I’ve had people say stuff to me in the grocery store or staring at him.” Caregivers felt like their communities were more “open and understanding” compared to outside of the communities. They also felt that there was more acceptance based on a cultural difference. As one caregiver put it, “I find that Cree, that my people are way more open and accepting,” and how they were “welcomed with such open arms.” Another caregiver touched on the cultural differences and how, because autism is viewed as a gift, acceptance is encouraged:

I have heard different perspectives in terms of like, being gifted, stuff like that. So, I think from a cultural perspective, what would you say? What, I appreciate it’s viewed in a more positive light than it is negative and there’s more of an acceptance to that difference, if you will, versus stigmatizing it in a negative way. To me, it’s been more of an acceptance. (P07)

Moreover, extended family acceptance was discussed. A caregiver shared, “My sisters, my mother-in-law didn’t treat him any differently than anybody else,” and when a child is seen as the same as the other kids in the family, it can create a space for them to be accepted in the family. Caregivers also felt their family’s acceptance of their child allowed them to accept their child as well, and “that acceptance that came from our family, I feel like that really did kind of help us remove that stigma.”

### Autism as a Gift

Despite challenges and concerns related to care for the child/adult with autism, caregivers described a different understanding of autism that included the cultural belief that autism is a gift. A caregiver shared,

There’s understanding of special needs, they’re very special children, they’ve been gifted, these are special children from the Creator. They’re more special than us. And that’s how they will just tell me and that I should be thankful and grateful for these special children to be my life.

Another caregiver discussed the “idea of really looking at the spiritual aspect and the specialness, the giftedness that these children and adults bring to the community. And like with my son, he’s very spiritual, and I support that.” Another caregiver shared,

[W]hen our kids are small, we’re told that they’re still connected to the Creator, and so everything that they say is really profound and I should be taken seriously, because they have more wisdom that says add up to have kind of lost that connection.

Although not all caregivers were aware of cultural understandings of autism, they still viewed autism as something to be “embraced” and “a learning opportunity.”

## Comparative Observations: Maskwacîs and Six Nations of the Grand River

Caregivers across both communities spoke about perspectives that were at times similar and at other times distinct. One of the most consistent themes was the barriers to culture. Many caregivers described feeling disconnected from their cultural roots, often expressing uncertainty about whether they and their children would be welcomed at cultural events. Some, however, shared that when they did attend cultural activities, such as longhouse gatherings in Six Nations or ceremonies in Maskwacîs, they noticed that these spaces were not always set up in ways that were inclusive of Autistic children. Expectations such as sitting still for long periods, enduring loud noises, or remaining quiet as a family created additional challenges.

Caregivers in both communities spoke about the compounding effects of ableism and racism, describing how they frequently experienced both on and off reserve. This dual burden made caregiving more difficult and often left them feeling isolated or marginalized. Another shared experience was within the school system. Many caregivers voiced concerns about bullying and negative attitudes from some staff, yet they also acknowledged positive experiences when their child had supportive teachers or when the child was able to excel in certain programs. These reflections highlighted both the struggles and the possibilities that caregivers navigate daily in supporting their children from two separate First Nations.

Across both communities, caregivers’ accounts showed that barriers were interconnected across systems. Diagnostic delays affected school supports; school exclusion intensified caregiver burnout; lack of on-reserve services forced some families to travel or relocate; relocation could weaken kinship support; and racism or ableism in health, education, and public spaces reduced caregivers’ trust in formal systems. These experiences demonstrate that families faced interconnected service problems rooted in colonial policy, jurisdictional fragmentation, and disability-related inequity.

Some of the key differences between caregivers from each community included the types of services they were expected to access. In Ontario, where Six Nations is located, most caregivers had accessed applied behavior analysis ABA therapy, whereas no caregivers accessed ABA in Maskwacîs in Alberta. This can be attributed to ABA therapy being a provincial mandate of the Ontario government. Overall, caregivers in the Six Nations mentioned currently accessing services, whereas Maskwacîs caregivers talked about wanting to access services but had more barriers due to being in a rural location. Maskwacîs caregivers (*n* = 4) also spoke about how they were providing foster care to their children, whereas in the Six Nations none of the caregivers were foster or adoptive care providers. There were also differences between the communities when examining Haudenosaunee in Six Nations and nêhiyaw understandings of autism in Maskwacîs. Finally,Maskwacîs parents often talked about cultural understandings such as autism as a gift, whereas these understandings were spoken about less in Six Nations.

## Discussion

Our findings resonate with the experiences of Indigenous caregivers globally. For example, a study examining the experiences of Māori whānau (family) raising Autistic children identified key caregiving priorities that included cultural goals, early childhood education, communication, and social values (Tupou et al., 2023). A strong desire for children to be raised within the cultural frameworks of te reo Māori and tikanga Māori emerged as a central theme. While caregivers acknowledged the utility of learning English, the transmission of Indigenous language and values remained a central caregiver objective. Similar to our findings, Māori caregivers expressed frustration with systemic barriers, particularly long wait times and inadequate culturally informed autism services. Tupou et al. (2023) recommended improved access to culturally grounded services and emphasized the critical role of relationships in working with Māori communities, an insight that echoes the importance of relationality (wâhkôtowin) expressed by caregivers in our study.

The present findings extend Indigenous autism scholarship by offering caregiver narratives from two First Nations contexts in Canada. Building on previous Indigenous autism research across settler-colonial contexts, our findings suggest that culturally grounded autism support requires attention to service access and the fit between autism supports and Indigenous worldviews (Bailey & Arciuli, 2020; Gerlach et al., 2022; Simpson, 2021; Tupou et al., 2021, 2023). Caregivers sought diagnosis, school support, and clinical services, while also calling for autism to be understood through kinship, cultural identity, ceremony, and responsibilities to children. This contributes to autism and Indigenous disability scholarship by showing how neurodiversity can be understood through relational and spiritual frameworks rather than solely through impairment-based models (Bruno et al., 2025; Inman, 2019; Lindblom, 2014).

Caregivers in our study frequently emphasized the importance of connecting their children with culture as a foundational aspect of health and well-being. This aligns with existing literature demonstrating the positive impact of cultural continuity and language revitalization. For instance, two studies found that Indigenous language immersion had significant benefits for student well-being, while another research project identified identity, spirituality, and traditions as protective factors contributing to the resilience of First Nations youth (Snowshoe et al., 2017; Whalen et al., 2016); however, these studies did not focus specifically on Autistic youth. Caregivers in our study similarly believed that grounding their Autistic children in culture would support positive health and learning outcomes. They also identified cultural worldviews as a key factor in fostering community acceptance of neurodiversity. When a child is raised with traditional teachings, caregivers noted they come to embody values and behaviors consistent with Indigenous knowledge systems. Many sought opportunities for their children to engage in cultural practices, such as attending powwows or participating in ceremonies, as these were seen as vital to holistic wellness.

A key theoretical contribution of this study is the framing of autism through First Nations worldviews of relationality and giftedness. Caregivers described Autistic children as gifts from the Creator and as children with spiritual insight, specialness, and community responsibilities. This framing acknowledges challenges such as burnout, service gaps, racism, and school exclusion, while refusing to define Autistic children through deficit or burden. Through wâhkôtowin, autism is understood in relation to kinship, belonging, responsibility, Ancestors, land, language, and community.

Structural barriers should be understood as interconnected rather than separate. Diagnostic delays, limited local services, jurisdictional divides, racism, inconsistent school support, bullying, and the absence of culturally appropriate programming intensified one another. For some families, traveling or relocating for services improved clinical access but weakened kinship, language, ceremony, and family support. These barriers show how colonial jurisdictional arrangements, ableism, and racism collectively shape caregiving.

Caregiver burnout must be interpreted within this relational and structural context. Caregivers were exhausted by system navigation, advocacy, cultural brokerage, and protecting their children from racism and ableism. Kinship, extended family, Elders, schools, and community programs can buffer burnout, but when these supports are disrupted by relocation, service gaps, or colonial histories of family separation, caregiving can become isolating. Caregiver wellness, therefore, requires coordinated systems of collective care.

In addition, the broader literature addresses caregiving supports in ways that are similar to many of the themes in our research. Studies have explored caregiver burnout and exhaustion (Fong et al., 2024), advocacy roles (Stahmer et al., 2019), positive reframing of autism as a gift (Cook, 2019), experiences of ableism (Furr, 2023), and challenges in navigating school systems (Martin-Denham, 2022). While these studies bring attention to shared caregiving challenges across diverse contexts, what distinguishes our study is the centrality of Indigenous worldviews in shaping caregiver experiences. In our study, caregiver burnout and advocacy were also shaped by First Nations experiences with colonialism, jurisdictional fragmentation, racism, cultural safety and relational responsibilities. Caregivers in our study often described autism through a cultural lens, often viewing it as a gift from the Creator or as part of ancestral continuity, thereby reframing autism outside of deficit-based paradigms (Napier & Whiskeyjack, 2021). This perspective served as a source of strength, resilience, and identity for many families.

The findings further demonstrate the central role of family, culture, and community strengths in supporting Autistic children and their caregivers. Caregivers described family acceptance, cultural teachings, drumming, dancing, hunting, smudging, school-based cultural activities, and ceremony as meaningful supports. Culturally grounded supports can include sensory-aware ceremony, flexible participation in cultural gatherings, land-based learning, language exposure, Elder involvement, peer and family education, and schools that affirm both Indigenous and Autistic identity.

The study findings have practical implications for policy and practice. Culturally grounded autism supports should be developed with First Nations leadership and include Elders, caregivers, Autistic people, educators, clinicians, and community workers. Supports could include community-based autism navigators, culturally safe diagnostic pathways closer to home, sensory-aware cultural programming, kinship-based respite, anti-racism and anti-ableism training, and service plans that include cultural goals. Health, education, and policy systems should partner with First Nations communities to co-design services, share authority, fund local capacity, and address jurisdictional fragmentation.

## Limitations

This research was conducted within the context of nêhiyaw culture and reflects the unique worldviews, teachings, and relational frameworks of our community. It also included caregivers from a Haudenosaunee community, and the comparative findings must be interpreted with careful attention to the distinct cultural, political, and historical contexts of each participating Nation. While the findings offer insights that may resonate with other Indigenous groups, it is essential to avoid pan-Indigenizing diverse cultural teachings and experiences. Indigenous groups across Canada (e.g., including Sioux, Anishinaabe, Blackfoot, Mi’kmaq, Coast Salish, Dene, Inuit, Métis, and others) hold distinct worldviews, languages, and protocols that shape how autism is understood and experienced. Future research must be community-led and culturally grounded within the specific ontologies and epistemologies of each Nation. For families to meaningfully engage with and understand autism, teachings must emerge from within their own cultural contexts. While certain teachings may be valuable across contexts, it is equally important to acknowledge their limitations and avoid assuming universality.

A further limitation of this study is the relatively small sample size (*n* = 14). While initial conversations suggested a strong interest in the research, fewer individuals followed through with participation once interviews were scheduled. Despite efforts to recruit and accommodate participants in ways that were relational and respectful, ongoing hesitancies around research participation remain. This is particularly pronounced when the topic is autism, which continues to carry stigma and misunderstanding in many communities. Additional barriers may have included fear of involvement with child and family services, historical trauma related to research, and broader systemic distrust. Another limitation is that all children discussed were boys, which may reflect sampling limitations and/or the under-recognition of autism in girls and gender-diverse children. This pattern should be interpreted cautiously, given broader gendered diagnostic inequities. These realities solidify the importance of long-term relationship-building, cultural safety, and community-driven research processes that affirm self-determination, reduce fear, and foster trust. As we intentionally limited the collection of potentially identifying demographic information, we were not able to examine how caregiver experiences may have differed across social locations such as socioeconomic context, household composition, language fluency, or gender identity. As a result, the findings should be read as a relational and thematic account of shared caregiver experiences rather than as an analysis of variation across caregiver contexts.

## Conclusion

Caregivers offered rich insights into the complexities of supporting Autistic children in First Nations contexts. Thematic analysis revealed interconnected domains of struggle, resilience, and cultural meaning-making. Understanding these themes is vital for informing responsive, culturally grounded, and community-driven autism supports. This study shows the urgent need for researchers, policymakers, and service providers to act on these findings by co-developing programs, policies, and practices that honor First Nations knowledge systems and caregiving realities. In practice, this means supporting First Nations-governed autism services, strengthening culturally safe diagnostic pathways, funding local and land-based supports, training educators and clinicians to address racism and ableism, and respecting caregiver expertise in schools, clinics, and policy settings. Services must recognize culture, language, ceremony, kinship, and community acceptance as central components of autism support rather than peripheral considerations. Overall, this study adhered to community principles, addressed a critical gap in the literature, and offered insights into the needs and priorities of First Nation caregivers that can guide future work in this important area.
